# Caring for hearts and minds: a quality improvement approach to individualized developmental care in the cardiac intensive care unit

**DOI:** 10.3389/fped.2024.1384615

**Published:** 2024-04-09

**Authors:** Samantha C. Butler, Valerie Rofeberg, Melissa Smith-Parrish, Meena LaRonde, Dorothy J. Vittner, Sarah Goldberg, Valerie Bailey, Malika M. Weeks, Sarah McCowan, Katrina Severtson, Kerri Glowick, Christine M. Rachwal

**Affiliations:** ^1^ Department of Psychiatry and Behavioral Sciences, Boston Children’s Hospital, Boston, MA, United States; ^2^ Department of Psychiatry (Psychology), Harvard Medical School, Boston, MA, United States; ^3^ Department of Cardiology, Boston Children’s Hospital, Boston, MA, United States; ^4^ Department of Pediatrics, Monroe Carell Jr. Children’s Hospital at Vanderbilt, Nashville, TN, United States; ^5^ Egan School of Nursing and Health Studies, Fairfield University, Fairfield, CT, United States; ^6^ Connecticut Children's Medical Center, NICU, Hartford, CT, United States

**Keywords:** developmental care, NIDCAP, infant development, cardiac intensive care, quality improvement

## Abstract

**Introduction:**

Infants with congenital heart disease (CHD) are at high risk for developmental differences which can be explained by the cumulative effect of medical complications along with sequelae related to the hospital and environmental challenges. The intervention of individualized developmental care (IDC) minimizes the mismatch between the fragile newborn brain's expectations and the experiences of stress and pain inherent in the intensive care unit (ICU) environment.

**Methods:**

A multidisciplinary group of experts was assembled to implement quality improvement (QI) to increase the amount of IDC provided, using the Newborn Individualized Developmental Care and Assessment Program (NIDCAP), to newborn infants in the cardiac ICU. A Key Driver Diagram was created, PDSA cycles were implemented, baseline and ongoing measurements of IDC were collected, and interventions were provided.

**Results:**

We collected 357 NIDCAP audits of bedside IDC. Improvement over time was noted in the amount of IDC including use of appropriate lighting, sound management, and developmentally supportive infant bedding and clothing, as well as in promoting self-regulation, therapeutic positioning, and caregiving facilitation. The area of family participation and holding of infants in the CICU was the hardest to support change over time, especially with the most ill infants. Infants with increased medical complexity were less likely to receive IDC.

**Discussion:**

This multidisciplinary, evidence-based QI intervention demonstrated that the implementation of IDC in the NIDCAP model improved over time using bedside auditing of IDC.

## Introduction

Congenital Heart Disease (CHD) is the most common cause of infant mortality, affecting approximately 40,000 infants born each year in the United States. In the first twelve months, one in four infants with CHD will require medical, therapeutic, palliative, or reparative invasive interventions (1, 2). Infants with complex CHD present significant medical and emotional challenges for their family and caregivers in the Pediatric Cardiac Intensive Care Unit (CICU). Infants in the CICU with CHD are at high risk for hemodynamic instability, hemorrhage, arrhythmia, multi-organ dysfunction, infection, and prolonged sedation withdrawal. They often experience hypersensitivity and are easily overwhelmed, display challenges to regulation and state management, and growth and feeding related difficulties (3–5). Neurodevelopmental deficits are noted in preschool and school aged children, including developmental delays, learning disabilities, and behavior related problems. These challenges require special education and social and emotional services (2–9).

In the growing population of children with CHD, research has often focused on medical and neurodevelopmental outcomes, with a limited, more recent focus on prevention and intervention strategies for implementation in clinical settings. While some developmental differences and challenges seen in children with CHD are explained by the cumulative effect of CHD related medical complications, many sequelae are not easily explained by medical complications alone (10–12). While the care provided in the CICU is necessary for survival, negative environmental and tactile stimulation potentially contribute to adverse neurodevelopmental outcomes. These excessive and unexpected modes of stimulation induce frequent activation of stress responses that alter brain function and structure (10, 13, 14).

Individualized Developmental Care (IDC) is an intervention designed to minimize the mismatch between the fragile infant's brain expectations and the experiences of stress and pain inherent in the ICU environment. Newborn Individualized Developmental Care and Assessment Program (NIDCAP) is the only evidence-based model of IDC (15). NIDCAP has been proven to improve outcomes for premature and critically ill infants by enhancing brain structure and function, along with improving behavioral outcomes that endure beyond infancy and into school age. In addition, NIDCAP studies report benefits for medical parameters such as decreased length of stay, earlier oral feeding, and increased weight gain, along with increased family engagement at the bedside, attachment to their infant, and confidence in caregiving (11, 16–28). There is overwhelming evidence for the positive effects of NIDCAP on families and children in the ICU, but there is variability in the impact related to varying methods and intensity of IDC delivered (29–31).

In an ICU that provides NIDCAP, family members are regarded as their child's primary caregivers and are the central providers of ongoing support of their child's clinical and developmental wellbeing. NIDCAP promotes sleep by creating a calm and soothing environment by individualizing light, sound, and activity around the infant and family. Beyond modification of the physical environment, specially educated and emotionally available healthcare professionals (HCP) collaborate to promote family nurturing, respect, collaborations and partnerships; and thereby reduce the infant and family's stress (10, 12, 24, 32). This shifting of care from the typical task and discipline orientation to a relationship-based orientation is challenging and requires increased self-awareness of the professionals' role from doer to facilitator and nurturer of growth and development (12, 30, 33).

Recent publications have stressed the importance of IDC in the CICU (34–36); however, it appears that IDC practices are implemented to varying degrees (31, 37, 38). The extreme nature of the medical needs of the CICU population provide an extra layer of challenge with extremely precarious patients, life threatening procedures, perilous lines and tubes, postsurgical obstacles, medical complications, extremely long hospital stays, comorbid medical conditions, large age range for patients, and traumatized families. Despite such challenges, research has noted that education for the HCP, even in medically complex environments, can improve IDC practices in the ICU (39, 40).

This manuscript describes a multi-dimensional program that used quality improvement (QI) to bring evidence-based practice (EBP) of IDC into the CICU through the NIDCAP model with the goal to provide sustainable improvements in clinical care. Practical EBP strategies included creation of an IDC program and implementation of NIDCAP through workgroups, education, and QI science. Our assumption was that, if an IDC program was implemented effectively, the health care team would increase their ability to provide individualized developmentally appropriate care at the bedside. While others have attempted to implement IDC, an integral piece of NIDCAP is observing and interpreting the infant's behavior. This key component is often omitted in other versions of developmental care. We focused on following the NIDCAP Developmental Care Guidelines (41), educating on observing infant behavior and understanding behavior in the context of behavioral subsystems so that each HCP could modify the environment and caregiving to provide individualized support for each infant and family (42, 43). We also hypothesized that IDC would be more challenging to implement with our most critically ill infants such as those with complex medical needs requiring more specialized medical equipment. Specific IDC interventions were targeted to the most critically ill patients and supports provided for their families.

## Materials and methods

### Context

This QI project took place in the CICU at a large 415 bed tertiary care hospital with over 30 beds, over 200 bedside nurses, and a multidisciplinary healthcare team. The heart center performs approximately 1,400 surgeries a year. All infants admitted to the CICU are born outside of BCH. The CICU cares for patients of all ages.

The Institute for Healthcare Improvement's (IHI) Model for Improvement^1^ was followed along with Plan-Do-Study-Act (PDSA) cycles to facilitate implementation of the QI project. The sequential steps in the Model for Improvement included: forming an expert multidisciplinary team, setting concrete goals and specific aims, establishing measures of current state of practice, testing changes, implementing changes, and spreading changes (44).

**Figure 1:**
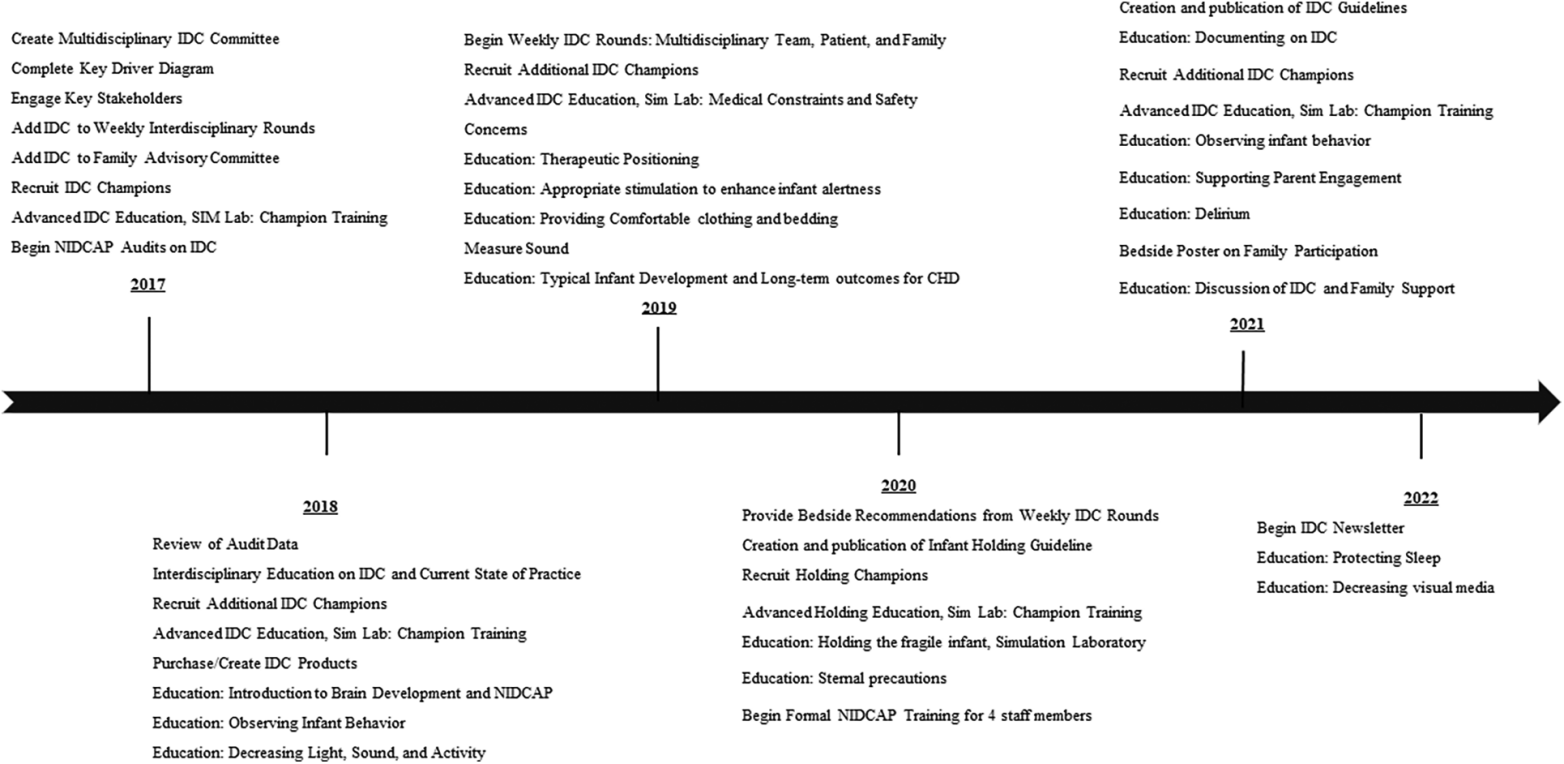
Timeline of quality improvement initiation and intervention of individualized developmental care.

**Figure 2:**
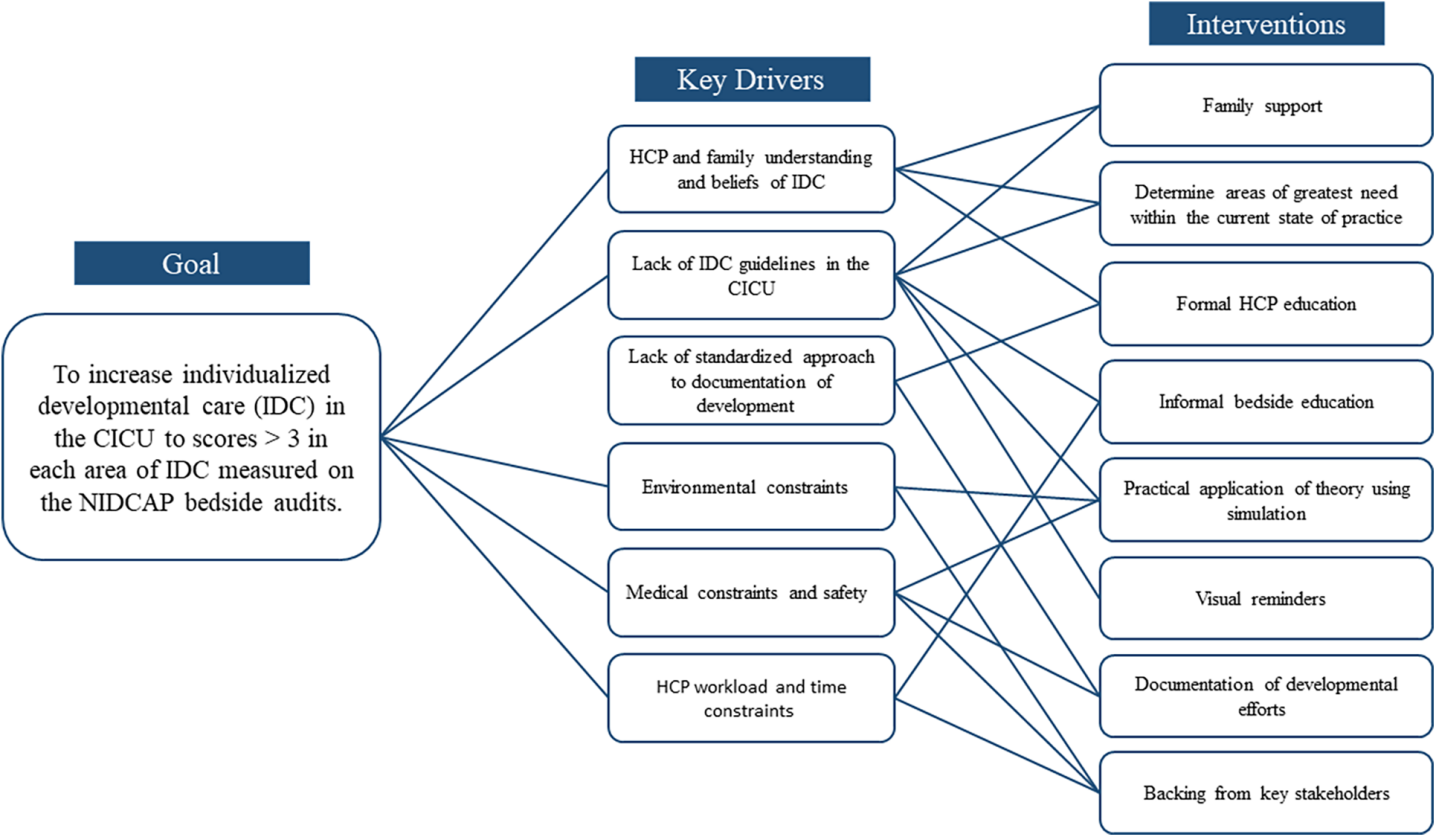
Key driver diagram.

QI methodology that was used included multiple PDSA cycles. A time line of study interventions is noted in Figure 1. PDSA cycle 1 consisted of creation of an IDC program in our CICU and a Key Driver Diagram (Figure 2). A multidisciplinary IDC Program was created in the CICU in 2017 and identified the importance of IDC and interest in a collaborative effort. The goal of the multidisciplinary team and IDC program was to provide education, policy implementation, family education, individual unit interventions and QI science on NIDCAP-based IDC. Routes toward implementation of IDC into the existing structure of the unit were discussed and agreed upon. The multidisciplinary team included psychologists, bedside nurses, advanced practice providers, physical and occupational therapists, music therapists, cardiac intensivists, cardiac surgeons, and respiratory therapists. The interdisciplinary nature of the working group allowed for collaboration across disciplines. The IDC program met monthly with an evening and morning meeting to include as many of the health care professionals working inpatient as possible during a given shift.

The Key Driver Diagram identified a specific aim of increasing individualized IDC in the CICU as measured using the NIDCAP bedside observational measurements. Key drivers included: (1) HCP understanding and beliefs regarding IDC, (2) family understanding and beliefs regarding IDC, (3) lack of standardized guidelines for IDC in the CICU, 4) lack of standardized approach to documentation of IDC, (4) environmental constraints, (5) medical constraints and safety concerns, and 6) workload and time constraints for providers. Interventions were created in response to the drivers. We determined that interventions that focused on gaining support from key stakeholders and measurement of current state of practice, providing education, and support for families and providers were necessary to improve IDC in the CICU.

During Cycle 2, Champions received additional education in the Simulation Laboratory on IDC and NIDCAP. IDC champions began data collection to measure the current state of IDC practice. Data was presented at monthly IDC program meetings. We also began presenting to unit wide management (in the fields of nursing, therapy, surgery, and cardiology) on the importance of IDC, supporting long-term developmental outcomes, and current state of practice.

Cycle 3 consisted of monthly review of data collection on the amount of bedside IDC provided for patients to identify educational gaps on the unit. Unit-wide education was created to address areas of concern. This was repeated several times until progress was seen in different IDC categories reviewed.

In Cycle 4, the IDC program completed a literature review and consensus opinions to create the standardized IDC guideline for the CICU. For Cycle 5, focus was directed to specific areas of improvement discovered after guideline release through data collection and analysis and further education was initiated. Focus on observing and interpreting infant behavior, providing care in response to infants expectations (cue based care), and providing IDC to our most ill patients was discussed and education provided over multiple educational roll outs.

### Measures

The primary process measurement included ongoing bedside auditing of IDC as a means of assessing current state of practice, understanding the impact of interventions over time, and assuring compliance with the NIDCAP model. NIDCAP Nursery Environmental Components Templates (NIDCAP audits) (45), were used to record the amount of IDC during a caregiving session and included: measurement of the physical environment of the nursery (Light and Sound), physical environment of the infant's bed space (Bedding and clothing), and specific aspects of direct infant care (Positioning, movement and tone, Timing and sequencing of caregiving interaction, Holding and caregiving facilitation, and Family participation). Each item was rated on a 5-point Likert scale ranging from 1 (traditional ICU care) to 5 (highly attuned individualized IDC in the NIDCAP model). Scores 3.5 and above demonstrate appropriate NIDCAP IDC.

The NIDCAP bedside observational audits were performed by trained IDC champions using a Redcap online program which facilitated electronic data collection and analysis. On training of the audits with IDC champions, auditors showed reliability with NIDCAP trainer of 90% before beginning individual audits. Information on demographics, medical background and intensity including: infant age, primary cardiac diagnosis, caregivers observed, type of care observed (medical intervention, medical emergency, routine caregiving, or other), medical devices in use (access and tubes) and sedative medications were collected. Our targeted population for the NIDCAP audits included infants between birth and 3 months of age receiving typical bedside care from any professional caregiver. We defined a 6 month baseline period prior to initiation of the interventions to improve care. We collected 15–20 NIDCAP audits per quarter which included both day and night shifts. Reviews of data collection feasibility, audit scores and data completeness were reviewed on an ongoing basis to help drive the PDSA cycles.

### Interventions

A variety of interventions (see Table 1) were implemented in response to the drivers and in response to audit data. As mentioned, monthly meetings included review of KDD and PDSA cycles. CICU guidelines were created following the NIDCAP IDC guidelines (41). The guidelines were verified by nursing leadership, cardiac surgery, and CICU leadership and were published in the internal hospital virtual library as a resource for multidisciplinary HCP.

**Table 1:** NIDCAP principles for IDC used for intervention.

NIDCAP IDC variables	IDC education provided	Specific IDC intervention
Light	• Diffuse dimmed light	• Observe behavior for individualized lighting • Low natural light when awake and alert • Darkness for sleep • Protect eyes from bright light
Sound	• Diffuse noxious sound	• Decrease voices, alarms, phones, music, and white noise • Talk to infant in soft voice • Provide soft family voices • Calm soothing atmosphere • Handle emergency situations in calm manner • Staff interactions away from bedside unless directly related to infant
Bedding and clothing	• Comfortable clothing and bedding	• Infant dressed • Observe behavior for individualized nesting/containment • Closeness to family • Space for family at bedside • Appropriate stimulation for infant alertness and interest
Specific support for self-regulation	• Appropriate stimulation to enhance infant alertness • Delirium • Decreasing visual Media • Documenting on IDC	• Four handed care (engaging help during caregiving from another individual) • Support into flexion • Containment • Soft voices • Pacifier or hand for sucking • Opportunities for hands to midline • Use of breast milk • Familiar faces • Observe infant behavior and provide supports as necessary
Position, movement and tone	• Therapeutic positioning • Sternal precautions	• Head in midline • Extremities flexed in midline • Hands available for grasping • Hands to face and mouth • Boundaries for containment and support for therapeutic positioning as necessary • Encourage supervised prone positioning • Holding in prone • Use of sidelying posture • Avoid hyperextension/arching/fisting
Timing and sequencing of caregiver interventions	• Observe infant behavior (determining organized/disorganized behavior) • Protect sleep	• Consider sleep-wake cycle • Time interventions • Support infant comfort throughout caregiving • Cluster and pace care based on infant behavior (cues of organized verse disorganized behavior) • Provide gentle touch
Holding and caregiving facilitation	• Holding the fragile infant	• In crib, snuggle with family hands • Family holding • Skin to skin holding,
Parent participation in care	• Parent engagement • Family support	• Family soft touch and engagement in caregiving early in admission

Modified from the NIDCAP Developmental Care Guidelines (41).

The largest area of intervention was HCP education. A Train-the-Trainer framework was used. Multidisciplinary Developmental Champions were educated as IDC subject matter content experts to enable training across the CICU. The NIDCAP professional and Nurse Practice Specialist provided a four-hour education module which focused on the understanding of long term developmental outcomes for individuals cared for in cardiology, brain development, individualized IDC, and NIDCAP research and implementation. Training culminated with hands-on practice in the simulation laboratory, highlighted by engaging with the simulator manikin to practice IDC strategies on a medically high risk infant. This developmental education program was presented to experienced nurses, PT/OT, respiratory therapy and intensivists and was a mandatory prerequisite to become an IDC champions. This education was repeated as new IDC Champions were recruited. Following education of the IDC champions, basic education on brain development and NIDCAP care was implemented unit wide to all nursing, intensivist and respiratory therapy teams.

Unit wide education was disseminated through a variety of avenues: Multimodal instruction was provided with PowerPoint lectures in small groups during nursing huddles, virtual learning modules were created, posters and emails were used for small informational blasts, and champions provided one to one bedside support for each bedside nurse. Specific areas of focus for education were directed by the audit results and areas of greatest need were noted.

IDC modules were presented in the yearly nursing education days along with presentations at staff meetings for cardiology, respiratory therapy, intensivists, nursing, and nurse practitioners. Additional materials to support education included: posters/signs around the unit to remind providers of IDC, an IDC newsletter for CICU providers, emails with latest research, and emails with quick reminders for practice. We also created a poster to hang in each patient room which highlighted for families the developmental objectives and areas of opportunity for family participation at the bedside.

In addition, IDC was incorporated into daily bedside rounds with the interdisciplinary medical team. This was initiated by nursing and supported by the intensivists. Separate weekly IDC rounds were also initiated in the CICU. IDC rounds included meeting at the bedside with the patient (if available), family members, bedside nurse, psychology, child life and other involved providers such as physical therapy, occupational therapy, feeding therapy, chaplaincy, social work, nutrition, music therapy, and lactation (10). The goal of these rounds was to provide infant assessment and family guidance, along with an opportunity to enhance the child's overall quality of care with interdisciplinary perspective. Education regarding the specific developmental needs of the patient with recommendations for interventions occurred in real time with the nurse(s) and other available medical team members at the bedside. Nurses and other team members were directly involved in the rounding process, both in learning key individualized developmental tools and also contributing pertinent information regarding clinical status.

IDC was integrated into the already standing Interdisciplinary Rounds which occurs once a week in a conference room for 30–60 min. It initially was created to review the medical status, social challenges, and provider needs for all patients in the CICU. Over time, the goals evolved to include patient developmental and educational concerns, environmental stressors, and family support. These rounds include bedside nursing, nursing leadership, child life, social work, music therapy, physical therapy, occupational therapy, psychology, chaplaincy, resource specialists, ethics, case managers, and intensivists. This exchange of information also allows for planning of co-treating across disciplines, coordination of care (scheduling therapies on different days), consistency of care (organizing a nursing team and primary physician), and the need for HCP support with challenging situations.

IDC products including beanbags and bed swaddles for supportive positioning and small pacifiers and swabs for delivering small amounts of breast milk were purchased. Custom supports were created including scent cloths for infant comfort and eye shields (dark colored felt cloth) that tented over the infant's eyes to protect from direct bright overhead lights and allowed the infant to see out during caregiving, When possible, indirect lighting was encouraged and utilized by HCP to complete caregiving tasks which was a shift in practice and unit culture.

### Analysis

Qualitative and quantitative analyses, along with data visualization tools, were used to draw inferences from the data. Process measures were the NIDCAP audit scores collected over time to quantify care practices. NIDCAP audit categories included: Light; Sound; Bedding and Clothing; Infant Self-Regulation; Position, Movement and Tone; Timing and Sequencing of Caregiving Interaction; Holding and Caregiving Facilitation; Family Participation in Care; and the overall average of NIDCAP audit categories. Shewhart control charts were used to assess improvement in NIDCAP audit scores over time (46). Data are plotted in time order with a central line for the average score over time, an upper line for the upper control limit, and a lower line for the lower control limit. The individual NIDCAP audit categories (Light; Sound; Bedding and Clothing; Infant Self-Regulation; Position, Movement and Tone; Timing and Sequencing of Caregiving Interaction; Holding and Caregiving Facilitation; Family Participation in Care) are plotted with the average score per quarter. The NIDCAP Average represents the average of all the individual audit categories. The center line was shifted and improvement in the score was considered significant if there were six consecutive points above the centerline.

Exploratory univariable linear regression was used to identify significant predictors (*P* < 0.05) of IDC. Balancing measures hypothesized to be related to infant care were included in stepwise linear regression. Candidate variables included: type of caregiving observed (standard care *vs*. emergency procedure), infant age (≤1 month *vs.* older than 1 month), cardiac anatomy (two-ventricle *vs*. single-ventricle *vs*. other), ventilation status (no ventilation *vs*. non-invasive ventilation *vs*. invasive ventilation), number of medical lines and tubes, open chest, VAD/ECMO, timing of observation (day *vs*. night shift), and current use of sedation. Predictors with univariable *P* < 0.20 were then considered for backward stepwise regression with multivariable *P* < 0.05 required to remain in the final models. All univariable and multivariable regressions were adjusted for time. Analyses of data were conducted in Excel QI Macros and R version 4.3.0.

### Ethical considerations

This protocol was submitted to the Institutional Review Board (IRB) and Scientific Review Committee, who determined it was exempt from full IRB review due to QI methodology. Patient confidentiality was protected and no identifying information was collected. No risk was placed to HCPs or patients and families with IDC observations.

## Results

Process measures were collected from 2017 to 2022 and included 357 IDC bedside NIDCAP audits. Table 2 reports background caregiving and patient characteristics. Fifty five percent of observations were during the day shift, 86% occurred during routine caregiving, and 93% of observations were of bedside nursing. Fifty two percent were under one month of age, 29% had single ventricle heart disease, 50% required invasive ventilation by endotracheal tube or tracheostomy, 70% had two or more medical lines and tubes, and 78% were on sedating medications.

**Table 2:** Background patient and caregiving characteristics (*N* = 357).

Characteristic	*n* (%) or mean ± SD
Caregiving observed	
Night Shift (7pm–7am)	159 (45%)
Observed during: Emergency or intervention	48 (14%)
Routine caregiving	304 (86%)
Caregivers observed: Nursing	333 (93%)
Medical	19 (5%)
Therapy^a^	34 (10%)
Family	51 (14%)
Other	13 (4%)
Patient factors	
Age ≤1 month	184 (52%)
Anatomy: Single ventricle	105 (29%)
Two ventricle	234 (66%)
Other (no structural heart disease)	17 (5%)
Ventilation: Invasive^b^	176 (50%)
Non-invasive^c^	20 (6%)
No ventilation	156 (44%)
Number of medical lines and tubes^d^: 0	25 (7%)
1	83 (23%)
2	118 (33%)
3	82 (23%)
4	36 (10%)
5	13 (4%)
Open Chest	10 (3%)
VAD or ECMO	4 (1%)
Sedation	269 (78%)

^a^ Includes occupational, physical, respiratory, music or feeding therapy.

^b^ Includes ventilation with endotracheal tube or tracheostomy.

^c^ Includes high flow nasal cannula, positive airway pressure (CPAP or BIPAP).

^d^ Includes arterial lines, central/umbilical venous lines, central catheters, peripheral intravenous lines, pacing wires, EEG, chest tubes.

Less than 10% missing from all variables.

**Figure 3:**
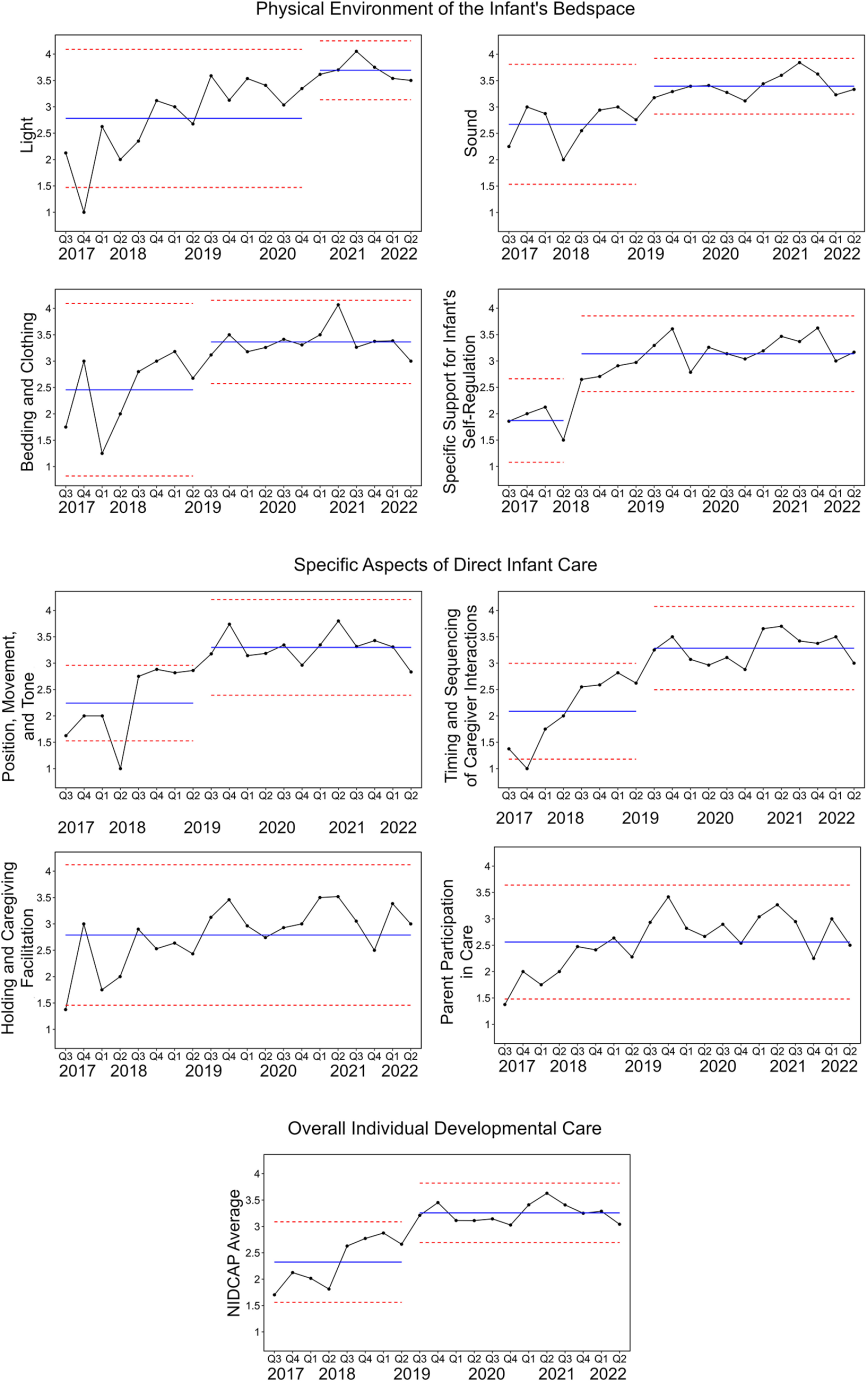
Control charts of NIDCAP individualized developmental care.

Control charts for each of the NIDCAP audit categories are shown in Figure 3. The categories of: Light; Sound; Bedding and Clothing; Infant Self-Regulation; Position, Movement, and Tone; Timing and Sequencing of Caregiving Interactions; and the overall NIDCAP average all showed significant improvement over time, with shifted center lines ranging from 3.1 to 3.7. The overall NIDCAP average of IDC care across all categories shifted from 2.3 to 3.3. The categories of Holding/Caregiving Facilitation and Parent Participation in Care did not demonstrate significant improvement, with center lines of 2.8 and 2.6 respectively.

Associations between NIDCAP audit categories with caregiving and patient factors are summarized in Table 3. Across all categories after stepwise regression, observation during an emergency procedure and presence of invasive ventilation were both independently associated with lower NIDCAP scores in all NIDCAP categories and in the NIDCAP average score (all *P* < 0.05). Audit during the night shift was associated with lower scores in all categories except Light, Sound, and Parent Participation in Care. Younger infant age at observation was associated with lower scores in all categories except Sound. An increased number of medical lines and tubes was associated with a lower Holding and Caregiving Facilitation score (*P* = 0.002), while having an open chest was associated with a lower Timing and Sequencing of Caregiving Interaction score (*P* = 0.003). Infants on sedation medication had lower Light (*P* = 0.001), and overall NIDCAP average (*P* = 0.01) scores.

**Table 3:** Univariate and multivariate analyses of NIDCAP audit category with background patient and caregiving characteristics.

NIDCAP audit category	Background and caregiving characteristics	Univariable beta [95% CI]	*P*-value	Multivariable beta [95% CI]	*P*-value
Light	Night shift	0.1 [−0.1, 0.4]	0.33	–	–
	Observed emergency procedure	−0.6 [−0.9, −0.2]	**0** **.** **001**	−0.5 [−0.8, −0.2]	**0** **.** **003**
	Age ≤1 month	−0.2 [−0.5, −0.01]	**0** **.** **04**	−0.3 [−0.6, −0.1]	**0** **.** **005**
	Single-ventricle anatomy	−0.3 [−0.5, −0.02]	**0** **.** **04**	–	–
	Other anatomy	0.0 [−0.5, 0.6]	0.94	–	–
	Non-invasive ventilation	0.0 [−0.5, 0.6]	0.90	0.1 [−0.5, 0.6]	0.79
	Invasive ventilation	−0.6 [−0.8, −0.4]	**<0** **.** **001**	−0.4 [−0.7, −0.2]	**0** **.** **001**
	Number of medical lines and tubes	−0.1 [−0.2, −0.05]	**0** **.** **003**	–	–
	Open chest	−1.0 [−1.7, −0.3]	**0** **.** **005**	–	–
	VAD or ECMO	−1.4 [−2.5, −0.3]	**0** **.** **01**	–	–
	Sedation	−0.6 [−0.9, −0.3]	**<0** **.** **001**	−0.5 [−0.8, −0.2]	**0** **.** **001**
Sound	Night shift	−0.02 [−0.2, 0.2]	0.87	–	–
	Observed emergency procedure	−0.6 [−0.9, −0.3]	**<0** **.** **001**	−0.6 [−0.8, −0.3]	**<0** **.** **001**
	Age ≤1 month	−0.2 [−0.4, 0]	**0** **.** **05**	–	–
	Single-ventricle anatomy	−0.01 [−0.2, 0.2]	0.94	–	–
	Other anatomy	0.3 [−0.1, 0.8]	0.15	–	–
	Non-invasive ventilation	0.2 [−0.2, 0.6]	0.37	0.2 [−0.2, 0.6]	0.30
	Invasive ventilation	−0.4 [−0.6, −0.2]	**<0** **.** **001**	−0.4 [−0.6, −0.2]	**<0** **.** **001**
	Number of medical lines and tubes	−0.1 [−0.2, −0.05]	**0** **.** **002**	–	–
	Open chest	−0.4 [−1.0, 0.1]	0.15	–	–
	VAD or ECMO	−0.3 [−1.2, 0.6]	0.51	–	–
	Sedation	−0.2 [−0.4, 0.0]	0.11	–	–
Bedding and clothing	Night shift	−0.4 [−0.6, −0.2]	**<0** **.** **001**	−0.4 [−0.6, −0.2]	**<0** **.** **001**
	Observed emergency procedure	−0.7 [−1.0, −0.4]	**<0** **.** **001**	−0.5 [−0.8, −0.3]	**<0** **.** **001**
	Age ≤1 month	−0.2 [−0.4, 0.0]	**0** **.** **05**	−0.2 [−0.4, −0.02]	**0** **.** **03**
	Single-ventricle anatomy	−0.05 [−0.3, 0.2]	0.66	–	–
	Other anatomy	0.3 [−0.1, 0.8]	0.15	–	–
	Non-invasive ventilation	−0.5 [−0.9, −0.1]	**0**.**02**	−0.5 [−0.9, −0.04]	**0** **.** **03**
	Invasive ventilation	−0.6 [−0.8, −0.4]	**<0** **.** **001**	−0.6 [−0.8, −0.4]	**<0** **.** **001**
	Number of medical lines and tubes	−0.2 [−0.2, −0.1]	**<0** **.** **001**	–	–
	Open chest	−0.8 [−1.4, −0.3]	**0** **.** **01**	–	–
	VAD or ECMO	−0.6 [−1.5, 0.4]	0.24	–	–
	Sedation	−0.4 [−0.6, −0.1]	**0** **.** **003**	–	–
Infant self-regulation	Night shift	−0.3 [−0.5, −0.1]	**0** **.** **001**	−0.3 [−0.5, −0.2]	**<0** **.** **001**
	Observed emergency procedure	−0.4 [−0.6, −0.1]	**0** **.** **004**	−0.2 [−0.5, 0.0]	**0** **.** **05**
	Age ≤1 month	−0.3 [−0.5, −0.2]	**<0** **.** **001**	−0.3 [−0.5, −0.2]	**<0** **.** **001**
	Single-ventricle anatomy	−0.1 [−0.3, 0.1]	0.41	–	–
	Other anatomy	0.1 [−0.3, 0.5]	0.77	–	–
	Non-invasive ventilation	0.1 [−0.3, 0.5]	0.70	0.1 [−0.3, 0.5]	0.69
	Invasive ventilation	−0.3 [−0.5, −0.1]	**0** **.** **001**	−0.3 [−0.5, −0.2]	**<0** **.** **001**
	Number of medical lines and tubes	−0.1 [−0.2, −0.03]	**0** **.** **004**	–	–
	Open chest	−0.3 [−0.8, 0.2]	0.30	–	–
	VAD or ECMO	0.1 [−0.7, 0.9]	0.80	–	–
	Sedation	−0.2 [−0.4, 0.04]	0.10	–	–
Position, movement, and tone	Night shift	−0.4 [−0.6, −0.2]	**<0** **.** **001**	−0.4 [−0.6, −0.2]	**<0** **.** **001**
	Observed emergency procedure	−0.9 [−1.1, −0.6]	**<0** **.** **001**	−0.7 [−0.9, −0.4]	**<0** **.** **001**
	Age ≤1 month	−0.3 [−0.5, −0.1]	**0** **.** **004**	−0.3 [−0.5, −0.1]	**0** **.** **001**
	Single-ventricle anatomy	−0.1 [−0.4, 0.1]	0.25	–	–
	Other anatomy	0.4 [−0.1, 0.8]	0.14	–	–
	Non-invasive ventilation	−0.2 [−0.7, 0.2]	0.29	−0.3 [−0.7, 0.1]	0.18
	Invasive ventilation	−0.5 [−0.8, −0.3]	**<0** **.** **001**	−0.6 [−0.8, −0.4]	**<0** **.** **001**
	Number of medical lines and tubes	−0.2 [−0.2, −0.1]	**<0** **.** **001**	–	–
	Open chest	−0.5 [−1.1, 0.2]	0.14	–	–
	VAD or ECMO	0.0 [−1.0, 1.0]	0.97	–	–
	Sedation	−0.4 [−0.6, −0.1]	**0**.**004**	–	–
Timing and sequencing of caregiving interaction	Night shift	−0.3 [−0.5, −0.1]	**0** **.** **01**	−0.3 [−0.5, −0.1]	**<0** **.** **001**
	Observed emergency procedure	−0.8 [−1.1, −0.5]	**<0** **.** **001**	−0.6 [−0.9, −0.3]	**<0** **.** **001**
	Age ≤1 month	−0.3 [−0.5, −0.1]	**0** **.** **003**	−0.3 [−0.5, −0.1]	**<0** **.** **001**
	Single-ventricle anatomy	−0.1 [−0.3, 0.1]	0.38	–	–
	Other anatomy	0.2 [−0.3, 0.7]	0.36	–	–
	Non-invasive ventilation	−0.4 [−0.9, 0.0]	0.06	−0.4 [−0.8, 0.0]	**0** **.** **05**
	Invasive ventilation	−0.6 [−0.8, −0.4]	**<0** **.** **001**	−0.6 [−0.8, −0.4]	**<0** **.** **001**
	Number of medical lines and tubes	−0.2 [−0.3, −0.1]	**<0** **.** **001**	–	–
	Open chest	−1.2 [−1.8, −0.6]	**<0** **.** **001**	−0.8 [−1.4, −0.3]	**0** **.** **003**
	VAD or ECMO	−0.4 [−1.4, 0.5]	0.38	–	–
	Sedation	−0.4 [−0.6, −0.1]	**0** **.** **01**	–	–
Holding and caregiving facilitation	Night shift	−0.2 [−0.4, 0.1]	0.19	−0.3 [−0.5, −0.1]	**0** **.** **004**
	Observed emergency procedure	−0.6 [−0.9, −0.3]	**<0** **.** **001**	−0.3 [−0.6, −0.1]	**0** **.** **01**
	Age ≤1 month	−0.4 [−0.6, −0.1]	**0** **.** **001**	−0.4 [−0.6, −0.2]	**<0** **.** **001**
	Single-ventricle anatomy	−0.1 [−0.4, 0.1]	0.35	–	–
	Other anatomy	0.4 [−0.1, 0.9]	0.15	–	–
	Non-invasive ventilation	−0.4 [−0.8, 0.0]	0.08	−0.3 [−0.7, 0.1]	0.13
	Invasive ventilation	−1.2 [−1.4, −1.0]	**<0** **.** **001**	−1.0 [−1.3, −0.8]	**<0** **.** **001**
	Number of medical lines and tubes	−0.3 [−0.4, −0.3]	**<0** **.** **001**	−0.1 [−0.2, −0.1]	**0** **.** **002**
	Open chest	−1.0 [−1.6, −0.3]	**0** **.** **005**	–	–
	VAD or ECMO	0.2 [−0.8, 1.3]	0.67	–	–
	Sedation	−0.6 [−0.9, −0.3]	**<0** **.** **001**	–	–
Parent participation in care	Night shift	−0.03 [−0.3, 0.2]	0.78	–	–
	Observed emergency procedure	−0.4 [−0.7, −0.04]	**0** **.** **03**	–	–
	Age ≤1 month	−0.3 [−0.5, −0.1]	**0** **.** **003**	−0.4 [−0.6, −0.2]	**<0** **.** **001**
	Single-ventricle anatomy	−0.2 [−0.4, 0.0]	0.10	–	–
	Other anatomy	0.3 [−0.2, 0.8]	0.21	–	–
	Non-invasive ventilation	−0.4 [−0.9, 0.1]	0.09	−0.4 [−0.9, 0.0]	0.06
	Invasive ventilation	−0.8 [−1.0, −0.6]	**<0** **.** **001**	−0.8 [−1.0, −0.6]	**<0** **.** **001**
	Number of medical lines and tubes	−0.2 [−0.3, −0.1]	**<0** **.** **001**	–	–
	Open chest	−0.4 [−1.1, 0.3]	0.24	–	–
	VAD or ECMO	0.4 [−0.6, 1.5]	0.39	–	–
	Sedation	−0.5 [−0.7, −0.2]	**<0** **.** **001**	–	–
NIDCAP average	Night shift	−0.2 [−0.3, −0.03]	**0** **.** **02**	−0.2 [−0.4, −0.1]	**<0** **.** **001**
	Observed emergency procedure	−0.6 [−0.8, −0.4]	**<0** **.** **001**	−0.5 [−0.6, −0.3]	**<0** **.** **001**
	Age ≤1 month	−0.3 [−0.4, −0.1]	**<0** **.** **001**	−0.3 [−0.4, −0.2]	**<0** **.** **001**
	Single-ventricle anatomy	−0.1 [−0.3, 0.0]	0.15	–	–
	Other anatomy	0.3 [−0.1, 0.6]	0.15	–	–
	Non-invasive ventilation	−0.2 [−0.5, 0.1]	0.16	−0.2 [−0.5, 0.1]	0.19
	Invasive ventilation	−0.6 [−0.8, −0.5]	**<0** **.** **001**	−0.6 [−0.7, −0.4]	**<0** **.** **001**
	Number of medical lines and tubes	−0.2 [−0.2, −0.1]	**<0** **.** **001**	–	–
	Open chest	−0.7 [−1.1, −0.2]	**0** **.** **003**	–	–
	VAD or ECMO	−0.2 [−0.9, 0.5]	0.51	–	–
	Sedation	−0.4 [−0.6, −0.2]	**<0** **.** **001**	−0.2 [−0.4, −0.1]	**0** **.** **01**

Significant values in bold.

## Discussion

In this quality improvement study, we explored the significant impact of IDC in the CICU for infants with CHD. Our approach utilized the NIDCAP model to bridge the gap between the infants' developmental needs and the complex intensive care environment. This multi-dimensional program of practice change indicated that it was feasible to measure the amount of NIDCAP care at the bedside for an individual patient. We also found a substantial improvement in the application of NIDCAP practices over time with ongoing intervention. Intervention was multimodally accomplished through in-person lectures, one-on-one education, simulation and literature.

Change in caregiving was evident in the use of more appropriate individualized lighting, sound management, and developmentally supportive infant bedding and clothing, as well as in promoting self-regulation and therapeutic positioning. For example, in measurement of light, NIDCAP audit scores indicated a change from baseline care where the environment was often bright with fluorescent overhead lights to the use of indirect lighting when infant was alert, providing darkness for sleep, and protection of infant eyes when light was needed. Baseline sound measurement indicated that loud human voices with background environmental noise was present much of the time in the infant's bedspace, however diminished sound scores were noted in the infant's care space post intervention. Developmentally supportive infant bedding and clothing improved from baseline and was individualized to the infant's preferences in order to support regulation (for example, gentle swaddling, soft hat/socks, appropriately fitting diapers, soft well-fitting clothing, and soft blankets). Assistance with infant self-regulation also improved over time. Prior to intervention, there was sporadic infant self-regulation support, following education HCP's consistently provided containment, gentle human touch, swaddling, and pacifiers to promote sucking. The promotion of therapeutic positioning to support appropriate musculoskeletal development, regulation, and infant comfort also increased and included the incorporation of supports (blanket boundaries, gel pillows, developmental positioning items) that maintained a well-aligned position in flexion and well-modulated tone. Timing and sequencing of caregiving interactions became increasingly sequenced to promote sleep, cluster care and support a paced approach to caregiving. Previously, care giving was more fragmented and in many cases implemented with inconsistent consideration of the infant's state and level of organization.

Data demonstrates that implementing family holding and increasing family involvement in infant caregiving proved challenging. There was less change seen over time, despite education and HCP support for these measures. Prior to the intervention, family holding and engagement with care was inconsistent. Post intervention, these data points demonstrated an increase in developmentally appropriate care but not enough to shift the center line on control charts and indicate a substantial change.

Findings illustrated that those infants with more complex medical needs were less likely to receive IDC. More specifically, infants receiving invasive ventilation, observed during emergency care, with increased number of medical lines and tubes, on sedation, younger in age, or with open chests were less likely to receive IDC. Overall, our data and QI process suggests that when a child is critically ill or medically complex, it is more complicated to provide IDC and that these areas of care need to be more thoroughly addressed in education for HCP. It was also noted that during the night shift, some areas of IDC were lower than during the day. Many of the IDC champions, including therapists and child life, are less available on the evening shifts which likely impacts IDC delivery.

Regardless of the varying impact of care across medical illness, the current evidence suggest that NIDCAP care and strategies were adaptable to a Cardiac ICU. Implementation in this highly medical environment was possible and demonstrates potential for sustained practice change and improvement. The implications of these findings are profound for future pediatric care in intensive settings. Not only does the study reinforce the importance of environment-sensitive care for vulnerable infants, but it also sets a precedent for the integration of developmental considerations into standard medical protocols. This holistic approach could potentially enhance long-term neurodevelopmental outcomes for infants in all ICUs.

The current QI study faces a few limitations. For example, we noted the challenge of consistently implementing IDC practices across varying medical complexities and in the face of operational constraints. Implementing IDC practices in a CICU requires a thoughtful and well-planned process to ensure successful adoption of practice changes with consistent support from departmental and institutional leadership. Research has shown that provider coordination is a large obstacle to IDC (47). The current study focused on multidisciplinary collaboration in order to increase adoption of care practice across the unit. Future studies should investigate the ability to provide consistent IDC for even the most ill patients. The current study also did not collect information on the caregivers' experience in the CICU or with IDC as it might be that some HCPs were more skilled at IDC than others, even prior to the QI study. Our study also took place during the global COVID pandemic which may have had a confounding effect on specific outcomes. It is possible that while we did not see an increase in parent engagement at the bedside during this QI study, we were able to maintain our current rate of parent participation even during the pandemic. In response to these findings our next QI project will look at increasing parent engagement at the bedside and holding of critically ill infants.

In sum, this multidisciplinary, evidence-based QI intervention demonstrated that the implementation of IDC improved overtime using a multimodal educational approach with bedside auditing to detect change in the CICU. With current literature advocating for IDC, this study demonstrates that it is possible to provide IDC in the NIDCAP model in the CICU. Of note, our study focused on all aspects of IDC including observing and responding to infant behavior which was shown improve overtime and lead to enhanced developmental care delivery. For future research, we propose a separate QI initiative targeting infant holding, parent bedside engagement, and supports for HCP working off shifts, all in promotion of infant well-being in the hospital. Additionally, a more extensive investigation into the long-term developmental outcomes of infants who received NIDCAP IDC in the CICU is needed. This would involve a longitudinal randomized study design, tracking developmental milestones and neurodevelopmental assessments over several years to fully understand the impact of early IDC interventions. A more robust understanding of the impact of IDC on infants with CHD would help advocate for system change in the CICU to include more NIDCAP based IDC.

## Data Availability

The dataset presented in this article is not readily available to outside institutions as it was intended only for our own quality improvement efforts and dissemination of final findings. As this was quality improvement, families and patients did not sign consent and data should not be shared without approval. Our patients and families trust us to keep their information secure and confidential. Allowing access to data collected may erode this trust and confidence, which is crucial for maintaining positive patient-provider relationships, especially in quality improvement studies. In summary, while using data for quality improvement in hospitals is essential for enhancing patient care and safety, it's equally important to ensure that this data is protected. Requests to access the datasets should be directed to the corresponding author.

## Footnotes

^1^ https://www.ihi.org/resources/tools/quality-improvement-essentials-toolkit
